# Sintering Analysis of Porous Ti/xTa Alloys Fabricated from Elemental Powders

**DOI:** 10.3390/ma15196548

**Published:** 2022-09-21

**Authors:** Rogelio Macias, Pedro Garnica-Gonzalez, Luis Olmos, Omar Jimenez, Jorge Chavez, Octavio Vazquez, Francisco Alvarado-Hernandez, Dante Arteaga

**Affiliations:** 1 Division de Estudios de Posgrado e Investigación, Tecnologico Nacional de Mexico/I.T. Morelia, Av. Tecnologico #1500, Colonia Lomas de Santiaguito, Morelia 58120, Mexico; 2 Instituto de Investigaciones en Ciencias de la Tierra, Universidad Michoacana de San Nicolas de Hidalgo, Morelia 58060, Mexico; 3 Departamento de Ingenieria de Proyectos, Universidad de Guadalajara, Zapopan 45100, Mexico; 4 Departamento de Ingeniería Mecánica Eléctrica, CUCEI, Universidad de Guadalajara, Blvd. Marcelino García Barragán #1421, Guadalajara 44430, Mexico; 5 Consejo Nacional de Ciencia y Tecnologia, Av. Insurgentes Sur #1582, Credito Constructor, Ciudad de Mexico 03940, Mexico; 6 Unidad Academica de Ingenieria I, Universidad Autonoma de Zacatecas, Zacatecas 98000, Mexico; 7 Centro de Geociencias, Universidad Nacional Autónoma de Mexico, Blvd. Juriquilla No. 3001, Queretaro 76230, Mexico

**Keywords:** sintering, microstructure, Ti-based composites, porous, microtomography

## Abstract

The present work is focused on developing Ti-xTa porous alloys processed by the space holder method and solid-state sintering. The volume fraction of Ta ranged between 20 and 30 wt.%. The sintering kinetics was evaluated by dilatometry tests. Sintered materials were characterized by SEM, XRD and computed tomography. Porosity features and permeability were determined from 3D images, and their mechanical properties were evaluated from microhardness and compression tests. The sintering behavior and the final microstructure are driven by the Ta diffusion into the Ti, slowing down the densification and modifying the transition temperature of *α*-to-*β*. Due to *β*-stabilization, martensite *α′* was obtained after sintering. Mechanical properties are reduced because of the *β*-stabilization and pore addition, being predominantly the pore effect. Permeability depended on the pore characteristics, finding values close to the human bones. It was concluded that powder metallurgy generates highly TixTa alloys with a combination of *α*, *β* and *α′* Ti phases as well as remaining Ta particles that are beneficial to improve the biocompatibility and osseointegration of such materials. Being the Ti25Ta40salt alloy the most suitable for orthopedic implants because of its characteristics and properties.

## 1. Introduction

Titanium (Ti) is a material with excellent mechanical and structural characteristics, such as high specific resistance and fracture toughness, fatigue, and crack propagation, as well as good corrosion resistance. Due to its relatively low density compared to other structural materials, titanium can be classified as a lightweight material. These properties, along with its good biocompatibility, make titanium a great choice for biomedical applications [1,2]. Nowadays, a lot of Ti alloys can be found, among them, the most used for orthopedic applications is the Ti-6Al-4V alloy, due to its low density, resistance to corrosion and relatively low stiffness [3]. There are different techniques to fabricate Ti alloys with complex geometries at low costs, such as Hot Pressing [3], Spark Plasma Sintering [4,5] selective laser melting [6], among others [7], leaving aside the conventional casting process. However, these techniques present an incomplete diffusion of alloying elements, namely Ta, Zr, Nb, Sn, Fe, Mo, Ni among others [8,9]. On the contrary, the conventional powder metallurgy (PM) technique is a process that helps to prevent this problem while allowing, to obtain complex parts at low costs, and with the possibility to control the mechanical properties through the sintering parameters [10,11]. In order to improve the titanium properties, the fabrication of titanium matrix composites (TMCs) has been largely developed as reported by JeJe et al. [12]. Most of the TMCs are developed to improve the mechanical properties, however, for biomedical applications this is not desirable. On the other hand, TMCs reinforced with hydroxyapatite were studied to successfully improve the osseointegration properties of the titanium and its alloys [13]. Notwithstanding the benefits of TMCs, their mechanical properties remain high in comparison to those of human bones.

The optimization of properties of Ti and its alloys depends on the control of the final microstructure and therefore, the stabilization of its phases such as *α*-Ti, *α* + *β* and *β*-Ti [5,9]. The mechanical properties of these alloys depend on the presence of several phases, which can be controlled by material processing and optimized alloy design [14]. It was reported a large strength improvement for bimodal grain size microstructure. Such characteristics can be obtained via powder metallurgy by mixing the initial particle sizes. This strengthening in pure Ti and Ti6Al4V alloy was due to a uniform distribution of the deformation between the fine and coarse grains, resulting in higher strength with a small decrement in the ductility [15,16]. The selection of Ti for implants application is determined by the combination of more favorable characteristics including corrosion resistance, low elastic modulus and superior biocompatibility. The lower elastic modulus in *β*-Ti alloys compared to stainless steel is a positive factor in reducing bone resorption [17]. *β*-Ti alloys have lower elastic modulus and present better formability and corrosion resistance than the *α*-Ti and *α* + *β* alloys [13,18,19]. Another advantage of *β*-Ti alloys is the possibility of the martensitic type transformations *β* → *α′* or *β* → *α″* between their metastable phases [13,20]. These alloys show low elastic modulus, shape memory behavior and super elasticity, which favors their applications in the biomedical field [9]. Due to that, research has been carried out on new *β*-Ti alloys whose microstructure can be tuned by the addition of different *β*-stabilizers elements [20,21].

Currently, one of the most suitable *β*-Ti forming materials for biomedical applications is Tantalum (Ta) since it has been shown to improve the biocompatibility and cytocompatibility of the Ti [22,23,24]. Furthermore, it was found that human stem cells can grow faster on a Ta surface than on a Ti surface, which is important for the patient recovery [25,26,27]. Ti-xTa alloys fabricated by several means including casting, selective laser melting and conventional sintering have been the target of some investigations due to their combination of properties such as high corrosion resistance and low elastics modulus [23,24]. It has been reported that the addition of Ta up to 30 wt.% generates a decrease in the elastic modulus, however, adding more Ta may cause the opposite effect [24]. The effect of Ta addition into a Ti matrix by the laser fusion technique has been investigated, it was found that the difference between the melting points of Ti and Ta, besides the relatively low diffusion coefficient of Ta, represent a difficulty when trying to completely combine both elements since Ta tends to segregate during solidification [24,25,26]. However, the study of the solid-state diffusion phenomena of Ti-Ta alloys by powder metallurgy has received less attention. The conventional PM method involving powder pressing and solid-state sintering remains one of the best techniques for producing complex parts with controlled mechanical properties [10,28]. In the PM technique, the possibility of using mixtures of elemental powders has great advantages in comparison with pre-alloyed powders, such as low costs, possibility of manufacturing in required complex shapes, and feasibility to combine the composition. Besides that, by means of PM, it is possible to control the elemental diffusion during sintering [29]. However, sintering conditions must be carefully controlled to avoid heterogeneous composition in the final parts.

In spite that Ta addition in Ti alloys significantly reduces the elastic modulus, it is still high in comparison to that of human bones, which generates the effect known as “stress-shielding”. To avoid these phenomena that leads to the failure of bone implants, highly porous materials called scaffolds have been developed [3,11,27,30]. A porous bone implant can appropriate consider some characteristics of biocompatibility, surface suitable to facilitate the adhesion of bone cells, an interconnected porosity for cell growth and transport of nutrients and finally the mechanical properties to meet the requirements of anatomical load avoiding the effect of stress-shielding [7]. Although additive manufacturing (AM) provides an excellent processing route to fabricate scaffolds, it is very expensive, and fabrication of Ti-Ta alloys is complicated because it implies the local fusion of particles. Thus, the space holder technique offers a less expensive route to obtain highly porous materials of Ti-Ta alloys, as it was shown by Garnica et al. that fabricated highly porous Ti6Al4V/xTa materials [31].

The aim of this work is to evaluate the addition of Ta particles into a pure Ti matrix and then, add different volume fractions of salt particles as space holders to create large pores. The sintering kinetics is determined by dilatometry tests and the resulting microstructure is observed by scanning electron microscopy (SEM) and X-ray diffraction. The distribution of the Ta and pores in the alloys is observed in 3D by X-ray microtomography. The mechanical properties of the different materials fabricated are evaluated by microhardness and compression tests.

## 2. Materials and Methods

### 2.1. Sample Preparation

**Figure 1:**
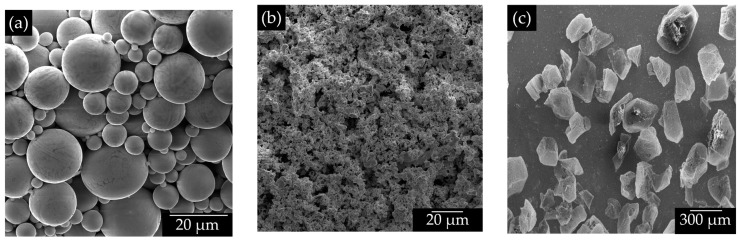
Micrographs of the used powders; (**a**) Ti; (**b**) Ta; and (**c**) ((NH_4_) HCO_3_).

Spherical Ti powders (Figure 1a) with particle size distribution less than 20 µm produced by Raymor, Quebec, Canada, were mixed with Ta powders (Figure 1b) of irregular shape and similar particle size distribution furnished by Sigma-Aldrich Co. (St. Louis, MO, USA), which were used as reinforcement. Irregularly shaped particles of ammonium bicarbonate ((NH_4_) HCO_3_) with a particle size distribution ranged 300–500 µm, furnished by Alfa Aesar, Tewksbury, MA, USA, were used as space holders to create large pores (Figure 1c).

Fabrication of samples consisted of two methodologies to generate parts with and without additional porosity. First, Ti powder was mixed in a turbula for 30 min in dry conditions with 20, 25 and 30 wt.% of Ta, respectively. Next, 1 wt.% of polyvinyl alcohol (PVA) was added as a binding for increasing the green compact resistance. After, the mixture was poured into an 8 mm diameter stainless steel die and pressed with a pressure of 500 MPa using an Instron 1150 universal machine to obtain cylindrical parts with approximately 12 mm height.

Likewise, in fabrication of samples with large pores, 30 and 40 vol.% of ammonium bicarbonate particles were added to the Ti-xTa mixtures and then, they were mixed in the turbula at the same conditions. After that, it was also added 1 wt.% of the PVA. Next, the same pressing procedure above described is followed. In order to eliminate the ammonium bicarbonate particles, the green compacts are introduced into a horizontal furnace at 180° during 4 h under Ar atmosphere before sintering.

Finally, all samples with or without additional porosity were sintered in a vertical dilatometer Lienseis L75V at 1260 °C with an isothermal time of 1 h maintaining a high purity argon atmosphere.

### 2.2. Microstructural Characterization

The sintered samples were cut and metallographically prepared by grinding and polishing with SiC paper and alumina suspension to achieve a mirror-like surface finish. The polished surfaces were subjected to X-ray diffraction tests (XRD) by using a PANalytical, Almelo, The Netherlands empirical diffractometer for the evaluation of the crystal structure obtained after sintering. The XRD standards were obtained using copper K alpha radiation with an energy of 30 kV and 30 mA, with a step of 0.2 and a time of 1 s per step in the range of 30–80°. Next, the polished surfaces were observed under a Tescan MIRA 3 LMU scanning electron microscope, SEM.

To evaluate the samples containing large pores, 3D images were acquired by computed microtomography (CMT) with a Zeiss Xradia 510 Versa 3D X-ray microscope. The beam intensity was 120 kV, which was enough to pass through Ti-xTa samples of 8 mm diameter. 1600 projections were recorded around 360° of the sample with a CCD camera of 1024 × 1024 pixels. The resulting voxel size was around 12 µm, which is enough to observe the whole sample. This resolution allows us to perform an analysis of the large pores created with the ammonium bicarbonate particles. Quantitative data of 3D images are obtained, after the initial gray level; images were transformed into binary ones by using manual thresholds constrained with the global relative density that was previously measured from mass and volume. In the obtained binary images, the solid phase represented by a voxel intensity of 255 and the pore phase by a voxel intensity of 0. This was achieved straightforwardly thanks to the strong contrast between both phases. Quantitative data of the porosity such as pore volume fraction, pore size distribution and channel size distribution were obtained following the image analysis explained elsewhere [32].

### 2.3. Permeability Evaluation

The flow properties of porous samples were determined by numerical simulations of permeability performed by using Avizo^®^ software version 2021 on the 3D binary images of samples with large pores. Before running the numerical simulations, the minimum representative volume (MRV) was defined by cropping the image in small cubes (20 × 20 × 20 voxels) at the center of the image and then, the relative density for that volume was calculated. These operations were repeated by increasing the size of the cube by 20 voxels per side until an almost constant relative density was reached, as proposed by Okuma et al. [33]. The minimal volume is useful to save time and computational requirements for the numerical simulations. It is found that the volume at which relative density reached almost a constant value is around, 250^3^ voxels^3^. A volume of 400 × 400 × 400 voxels, represents 110 mm^3^, was used for running the numerical simulations that were performed in the 3 main directions of the cube, were “*x*” and “*y*”, represents the radial plane and “*z*” the vertical axis of the cylinder.

Simulations on Avizo^®^ are based on the Darcy law by solving Navier Stokes equations with a finite volume method. The simulation considered a single-phase incompressible Newtonian fluid with a steady state laminar flow and a viscosity of 0.045 Pa, which represents the viscosity of the blood. The boundaries conditions used were the inlet and outlet pressure, with values of 130 and 100 kPa, respectively. The absolute permeability is computed with a single-phase flow. The module takes a labeled image as input, but each label of the image has to be part either of the solid or of the fluid phase: only one solid and one fluid phase are considered for this calculation. The solid phase is impermeable: there is no flow in it.

### 2.4. Mechanical Properties Evaluation

To evaluate the mechanical strength, compression tests were performed on the sintered samples according to ASTM D695–02 with an Instron 1150 universal testing machine with a head speed of 0.5 mm/min [10]. The elastic modulus (E) and the elastic limit (*σ_y_*) were estimated from the elastic stage of the stress-strain curves, after correction with respect to the displacement of the equipment.

## 3. Results and Discussion

### 3.1. Sintering Kinetics Analysis

**Figure 2:**
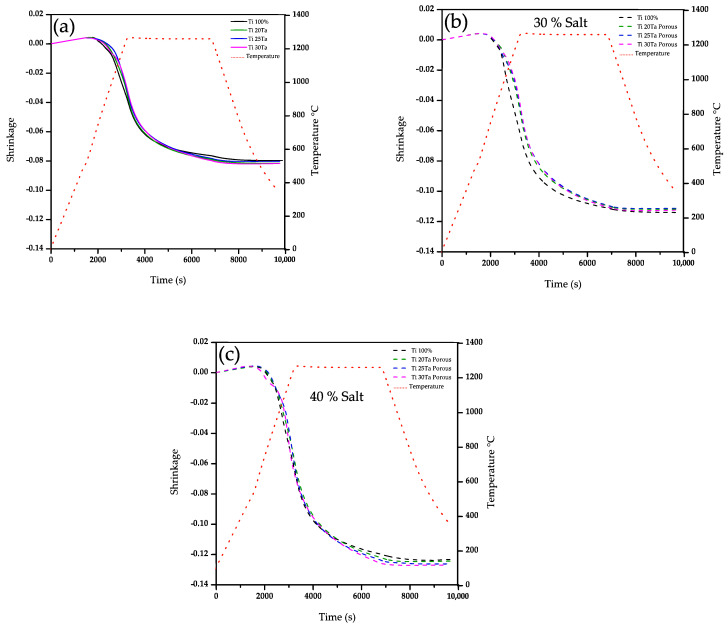
Strain as a function of temperature during the whole thermal cycle for (**a**) Ti-xTa samples; (**b**) Ti-xTa with 30 vol.% pore formers; and (**c**) Ti-xTa with 40 vol.% pore formers.

The axial strain as a function of the time and temperature during sintering of Ti and Ti-xTa compacts with and without large pores was plotted in Figure 2. All samples show an initial expansion due to the thermal expansion of the material. Next, there is a change in the slope of the strain becoming negative, which indicates the start of sintering at 613 °C. Next, there is an abrupt increase in the strain as the temperature increases until reaching the isothermal temperature, where an asymptotic behavior is observed during the sintering plateau. The maximum shrinkage reached for the Ti sample agrees with those reported by Panigrahi et al. for similar sintering conditions [34]. The addition of Ta in the Ti matrix generates a small increment in the shrinkage of 2.5% in comparison to that of Ti, Figure 2a. The addition of large pores enhanced the shrinkage obtained because the large pores are shrinking during sintering. It can be noticed that larger shrinkage is obtained as the volume fraction increased, Figure 2b,c. This because of the deformation of large pores induces an additional shrinkage due to densification stresses generated by sintering, as it was also reported for porous Ti6Al4V by Cabezas et al. [11]. Another point of view could suggest that greater contraction could be obtained because the presence of large pores improves the particle rearrangement generated by free movement of particles in the sintering process towards the periphery of these large pores as it was pointed out elsewhere [35].

**Figure 3:**
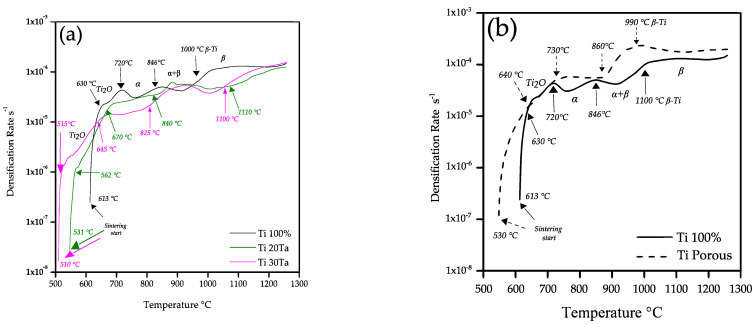
Densification rate as a function of temperature during heating up to 1260 °C of; (**a**) Ti and Ti-xTa; and (**b**) Ti without and with 30 vol.% of pore formers.

Figure 3a shows the densification rate as a function of temperature during heating stage for Ti-xTa samples. A first increment in the densification rate is found at 613, 531 and 510 °C for Ti, Ti-20Ta and Ti-30Ta, respectively. This is associated with the sintering activation. The difference in temperatures for the samples is because the Ta diffusion into the *α*-Ti matrix starts around 520 °C according to previous reports [35,36]. After that, a continuous increment in the densification rate is observed and during the heating stage can be detected two main changes in the trend of the densification rate. The first one is associated to the absorption of oxygen into the Ti atomic network at around 720 °C, as it was found elsewhere [37,38]. It is observed that Ta addition reduces the temperature at which the oxygen absorption is activated, 670 and 660 °C for 20 and 30 vol.% of Ta, respectively. It is also noticed a smaller increment in the densification rate, which suggest that lower quantity of oxygen can be absorbed because the Ta atoms are already in the Ti crystalline net. The second change in the trend of densification rate corresponds to the beginning of the phase transition *α* → *α* + *β* at 846 °C, which leads a complete transition to *β*-Ti phase at 1000 °C. Those temperatures agree with those reported for the transition phase of Ti solid [39,40]. The addition of Ta reduces the temperature of the transition phase *α* → *α* + *β* by up to 21 °C. Another effect generated by the addition of Ta is that the transition temperature in where both phases *α* and *β* coexist has an increment from 154 °C up to 275 °C by the time *β* is reached. This indicates that the addition of Ta causes a slow development of the transformation of *α* → *β* since the solubility of Ta in Ti starts to deteriorate from 3.6% of Ta at 550 °C for the solid state [39]. Figure 3b shows that the changes associated with the start of the phase transformation are not significant, which indicates that the content of pores in the matrix does not affect the phase change temperatures in the Ti matrix.

The density of all samples was calculated by weighing them and measuring their volume. The values of the green and sintered density for the different samples are listed in Table 1. It is observed that the presence of Ta as a high-density element (16.65 gr/cm^3^) generates an increment in the density values, as expected. The addition of 30 wt.% of Ta induces an increment of 22.7% with respect to the sample without Ta. The densities of samples containing 30 and 40 vol.% of pore formers are highly reduced, around 60% with respect to the same Ti-xTa sample without large pores. This is beneficial for bone implants applications since the density reported for trabecular bones ranged between 0.8 and 1 g/cm^3^ and compact bones ranges between 1.2 and 2 g/cm^3^ [41,42]. This means that samples with 40 vol.% of pores are in the range, which are also in the range of the optimal density value 1.8 g/cm^3^ suggested by Adamovic et al. for bone implants [43].

### 3.2. Microstructural Analysis

**Figure 4:**
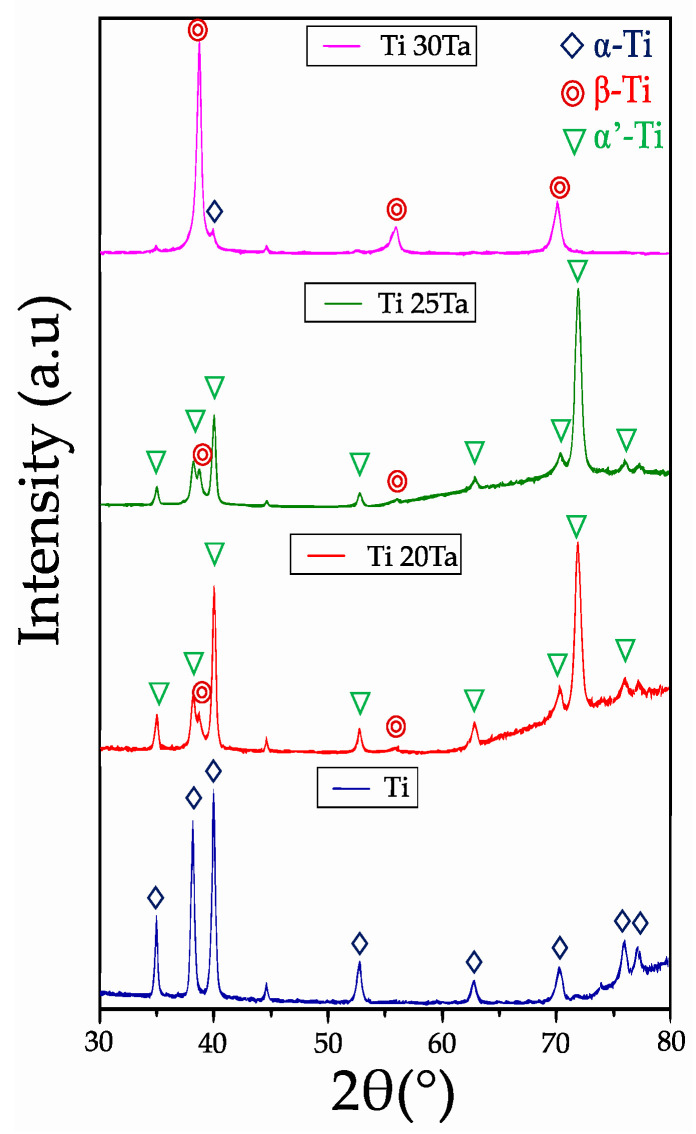
XRD patterns after sintering of Ti and composites with 20, 25 and 30 wt.% of Ta.

Figure 4 shows the X-ray diffraction patterns of Ti-xTa samples with 0, 20, 25 and 30 wt.% of Ta. The sample without Ta, only the characteristic peaks of the *α*-Ti phase are found as expected. The addition of Ta generates a change in the microstructure, first addition of 20 wt.% Ta promotes a small quantity of *β*-Ti phase since it can be detected in the main peak at 39.17°. It was also determined that additions of 20 and 25 wt.% Ta shows a slight displacement of the *α*-Ti peaks that suggest the formation of the martensite *α′*-Ti phase, which is also confirmed by the presence of a high peak at 72°. These characteristics were reported as an indicative of the presence of the martensite phase of titanium for other authors [23,44,45,46]. The addition of 30 wt.% of Ta generates a high increment in the intensity of the *β*-Ti phase characteristic peaks, being the predominant phase of the Ti30Ta alloy after sintering [47].

**Figure 5:**
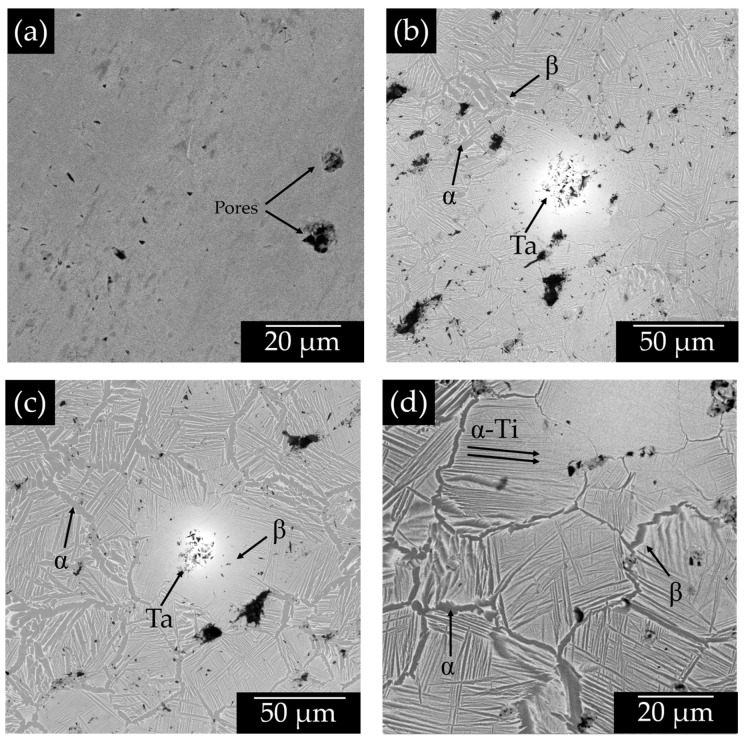
TSEM micrographs of the different Ti-xTa samples with (**a**) 0%; (**b**) 20 wt.%; (**c**) 25 wt.%; and (**d**) 30 wt.%.

SEM micrographs of samples with 0, 20, 20, 25, 30 wt.% of Ta are shown in Figure 5. It is observed that the sample without Ta shows some isolated spherical pores without the *β*-Ti lamellae (Figure 5a), which confirms the X-ray patterns discussed in Figure 4. As the Ta content in the matrix increases, it is easy to distinct zones of the *β*-Ti phase (Figure 5b) and lamellae of the *α*-Ti phase present. In addition, around the Ta particle a rich zone of *β*-Ti phase is present, indicating that this phase is more stable near Ta [24]. It is also observed that *α*-Ti phase is segregated at the grain boundaries of the Ti particles (Figure 5c). Furthermore, it is also appreciated that the *α*-Ti lamellae are thinner and with a more symmetrical orientation as the Ta content increases that is more obvious for the sample with 30 wt.% of Ta (Figure 5d). Similar microstructure was reported for Ti-Ta alloys fabricated by casting [46].

**Figure 6:**
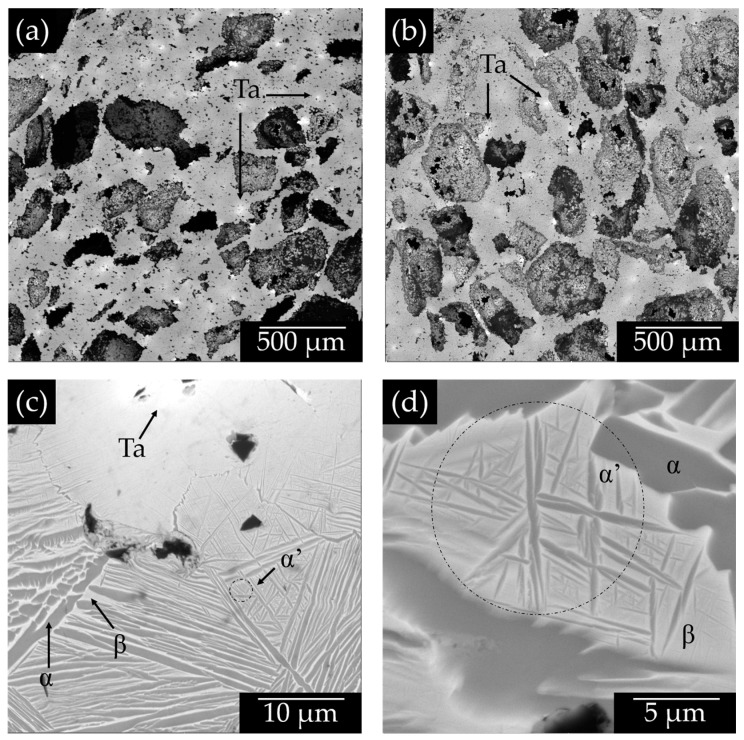
SEM micrographs of the different Ti-30Ta samples with large pores; (**a**,**b**) with 30 and 40 vol.% of pore formers added, respectively; and higher magnifications (**c**,**d**), respectively.

Figure 6a shows the sample with 30 wt.% of Ta and 30 vol.% of pore formers in which it is possible to observe a random distribution of large pores, which is similar for the sample containing 40 vol.% of pore formers, Figure 6b. It is noticed that the porosity does not have a significant change in the microstructure of the Ti-xTa alloys. Although some white dots close the pore boundaries indicates that Ta particles cannot completely diffuse into the Ti-net because they are not surrounded by Ti particles. In addition, it is noticed a homogeneously distributed of the remaining Ta particles in the Ti matrix, indicating that the fabrication methodology helps such distribution of Ta and pores. Figure 6c confirms that similar microstructure is developed in porous samples in comparison to the one found in samples without large pores, Figure 5d. Furthermore, the presence of small needles such as microstructure inside the *β*-Ti lamellae is detected (Figure 6d), which confirms the formation of the *α′*-Ti phase as it was discussed above by XRD analysis [45,46]. The martensite phase has a greater presence in the matrix for the highest Ta content, this phase has been obtained in Ti-Ta alloys produced by casting after quenching [45,48]. This could suggest that fully martensite could be obtained by quenching the sintered samples because the *β*-Ti is predominant in the microstructure. Moreover, it is found the presence of isolated spherical pores distributed in the matrix, indicating that the last stage of sintering was reached.

**Figure 7:**
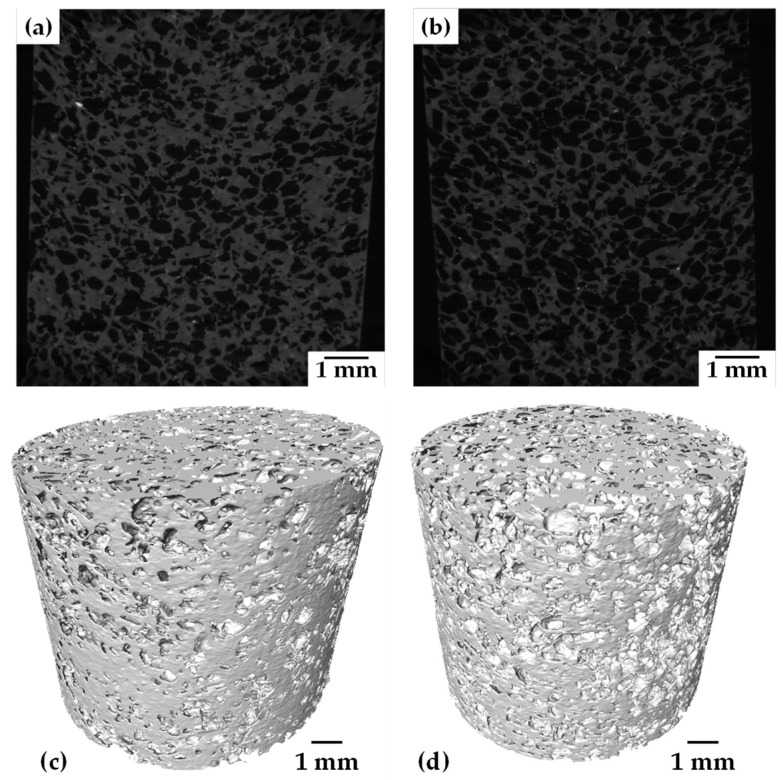
2D virtual slices and 3D rendering of: (**a**,**c**) Ti00020Ta 30 vol.% salt; and (**b**,**d**) Ti20Ta 40 vol.% salt.

**Figure 8:**
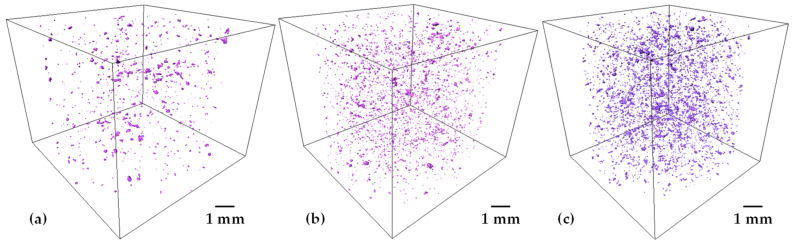
3D rendering of the remaining Ta particles after sintering for samples containing 30 vol.% of salt particles, (**a**) Ti20Ta; (**b**) Ti25Ta; and (**c**) Ti30Ta.

In order to have a tridimensional analysis of the added porosity, 3D images with a voxel resolution of 12 µm were acquired. 2D virtual slices of Ti20Ta-30salt and Ti20Ta-40salt samples are shown in Figure 7a,b, respectively. It is observed that large pores created by salt elimination are randomly distributed in the whole sample, sample with 40 vol.% of salt particles shows some agglomeration of pores that leads to the formation of larger pores, which is expected since the coordination between salt particles increases as the volume fraction does it. A 3D rendering of the complete cylinder of Ti20Ta-30salt and Ti20Ta-40salt samples is shown in Figure 7c,d, respectively. Qualitatively two main features can be distinguished in both kind of samples, the first one is that wall thicknesses between large pores is reduced as the volume fraction of salt particles increases. The second one is that Ta particles can be identified, white spots in Figure 7a,b, and they are well dispersed in the matrix, suggesting a good distribution of Ta particles. Quantitative data about the porosity, Ta particles and wall thicknesses were calculated from 3D images. For that, 3D images follow different mathematical operation to convert the initial grey levels images into the binary ones. The segmentation process is detailed elsewhere [31]. The remaining Ta particles after sintering in the samples with 20, 25 and 30 wt.% of Ta are shown in Figure 8. It is observed that the quantity of Ta particles increases as the Ta wt.% increases too, which suggest less diffusion of Ta into the matrix achieved during sintering. This could be because the contacts between Ti and Ta particles is reduced by the increment in Ta-Ta contacts, thus, diffusion of Ta into the matrix slowed down since it is driven in solid state. The volume fractions of remaining particles are 0.43, 0.85 and 1.55%, for the Ti20Ta, Ti25Ta and Ti30Ta samples, respectively. This indicates that most of Ta particles can diffuse into the Ti matrix. This could be due to the good distribution of Ta particles that do not show segregation neither agglomeration despite its density which is around 4 times with respect to Ti.

**Figure 9:**
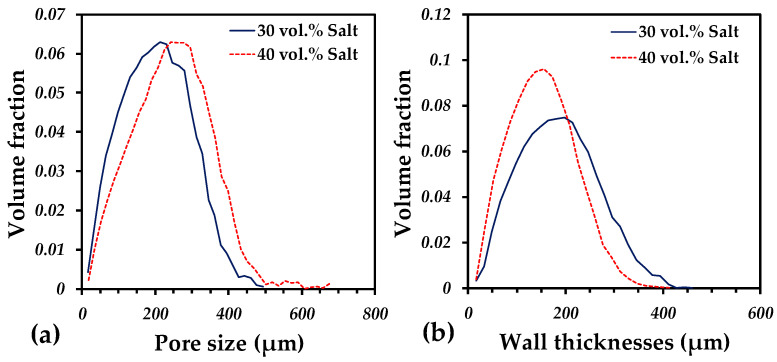
Pore size distribution (**a**) and wall thicknesses distribution; (**b**) for porous samples fabricated with 30 and 40 vol.% of salt particles.

The pore size distribution for samples with 30 and 40 vol.% of salt particles goes from pores of 40 µm to the largest ones around 650 µm, Figure 9a. It is found that pore sizes increase as the quantity of salt particles does it. This suggests that some particles can be grouped to form larger pores. This small increment generates that the median pore size increased from 195 to 224 µm, see Table 2. It is also noticed that pores up to 650 µm can be formed for 40 vol.% of salt particles, meanwhile the largest pore for the samples with 30 vol.% is 500 µm. This distribution is mainly due to the interparticle pores left after sintering and by the artificial porosity created by the salt elimination, which is good for the bone implant applications since the optimal pore size for bone ingrowth have been demonstrated for pores smaller than 10 µm [49], or pores larger than 900 µm [50]. Furthermore, Itälä et al. [51] considered that the optimal pore size range was 100–400 µm.

Another effect of the addition of pores is the wall thickness of the solid structure because there is more void space generated due to the salt elimination. The wall thicknesses distribution is obtained by the technique of granulometry in the same way that the pore size distribution and depicted in Figure 9b. It is found that the addition from 30 to 40 vol.% of salt particles reduced the median wall thickness around of 15%, from 175 to 150 µm, see Table 2. It is also observed that the quantity of wall thicknesses lower than 100 µm increases for the sample with 40 vol.% with respect to the 30 vol.% of salt particles added.

### 3.3. Permeability Analysis

**Figure 10:**
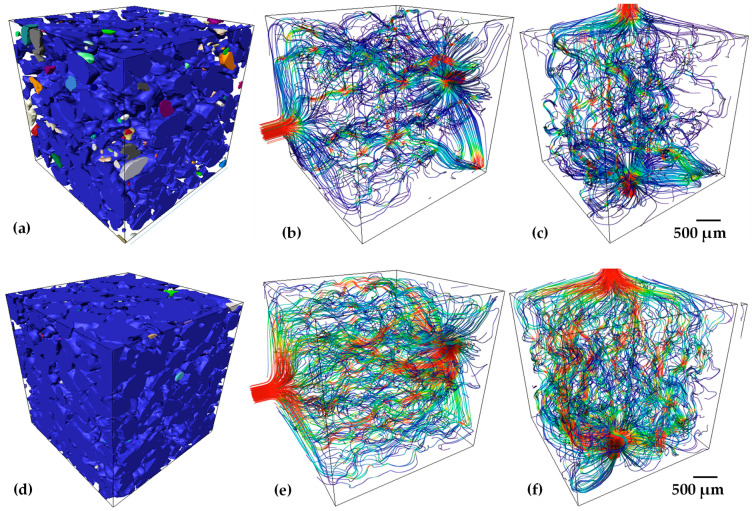
3D volume renderings of porosity, with colors depicting connected pores, and streamlines for the simulated flow of blood through the pores in the vertical and horizontal directions, with the colors depicting fluid velocity, for samples with 30 wt.% of Ta and either 30 vol.% (**a**–**c**) or 40 vol.%; (**d**–**f**) of salt particles.

In order to estimate the permeability of porous samples, numerical simulations on the 3D images obtained by tomography were performed by using the Avizo software. The numerical simulations of permeability were carried out in sub-volumes of 400 × 400 × 400 voxels, and 4 sub-volumes were extracted from different zones of samples. The average permeability value for samples with 30 and 40 vol.% of salt particles is listed in Table 2. It is found that permeability increases 10 times for the samples fabricated with 40 vol.% of salt particles with respect to the ones with 30 vol.%. This increment is due to different features of the porosity, first the volume fraction of pores increases from 42 to 58%. This is mainly because small agglomerates of salt particles appear, creating larger pores in comparison to 30 vol.% of salt particles used. In addition, the pore size increases 13% from 195 to 224 µm, which increases the permeability. Furthermore, as the pore volume increases, the pore connectivity does it. The connectivity can be qualitatively observed from 3D images of the labeled pores, Figure 10a,d. In such figures, the same color indicates that pores are connected, and a different color shows isolated pores. As it was expected, the sample with 30 vol.% of salt particles shows more isolated pores than the one with 40 vol.% that is almost fully connected. This can also be confirmed in the flow lines obtained from the numerical simulations that show more flow paths throughout the sample with 40 vol.%, Figure 10e,f, with respect to the sample with 30 vol.% of salt particles Figure 10b,c. The last feature that affects the permeability is the tortuosity, which is reduced 16% from 1.54 to 1.34 for the sample with 40 vol.% of salts, see Table 2. The flow streamlines indicate the velocity of fluid throughout the pore media in a color code, in which the blue means slow and red fast. This is a qualitatively indicative of the ability to let the fluid pass throughout the porous media, thus, the sample with 40 vol.% of salts shows faster velocity of the fluid than the sample with 30 vol.% of salts. The higher permeability value calculated of 1.15 × 10^−10^ m^2^ is close to the range reported for human bones, 3 × 10^−11^ to 5 × 10^−10^ m^2^ for human proximal femur and 10^−8^ to 10^−9^ m^2^ for human vertebral body [52].

**Figure 11:**
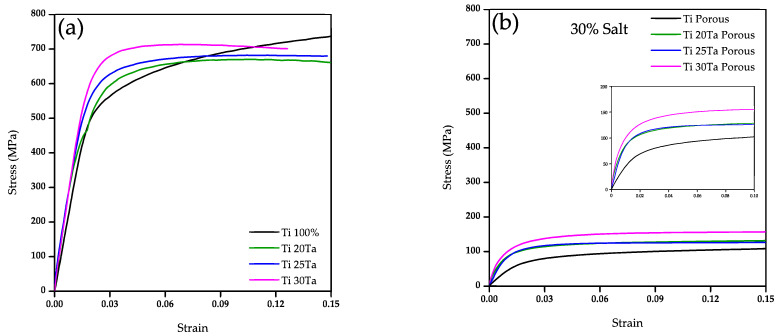

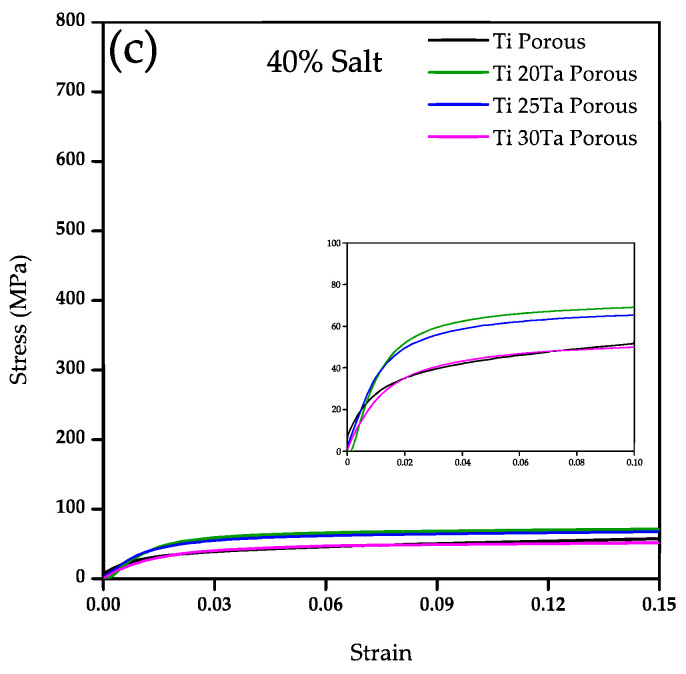
Stress-strain curves of samples sintered at 1260 °C, (**a**) Ti-xTa; (**b**) Ti-xTa with 30 vol.% of salt particles; and (**c**) Ti-xTa with 40 vol.% of salt particles.

The stress-strain curves of Ti-xTa alloys with and without additional porosity sintered at 1260 °C are shown in Figure 11. It is observed that the addition of Ta into the Ti increases the yield strength from 450 MPa for Ti to 580 MPa for the alloy with 30 wt.% of Ta, Figure 11a. Comparing the behavior of the curves, it is noticed that the stress in the Ti sample increases during the plastic deformation, which indicates a plastic hardening. On the contrary, samples with Ta addition shows a maximum value of the stress during the plastic deformation and then a plateau, which indicates a more ductility in the plastic region. The mechanical properties are due to the diffusion of Ta within the Ti matrix and the stabilization of the *β*-Ti phase [53]. Although, the strength of *α* and *β* phases has been also studied, and it is reported that the *β*-Ti phase has a lower elastic modulus than *α* [54,55,56,57]. The small increment found in this work could be due to the Ta particles cannot be fully diffused into the matrix, therefore, strengthening mechanisms of the matrix could increase the mechanical strength.

The compressive behavior of Ti-xTa alloys with 30 and 40 vol.% of additional porosity added is shown in Figure 11b,c, respectively. A sharp reduction in the mechanical strength is obtained by the addition of pores for all samples. The reduction in the yield stress for all samples is around 8 and 14 times for samples with 30 and 40 vol.% of salts, respectively. The behavior during the plastic deformation for all samples is similar with a maximum value of the stress and with a long plateau, which indicates a more ductility due to the addition of large pores that increases the deformation of the samples.

**Figure 12:**
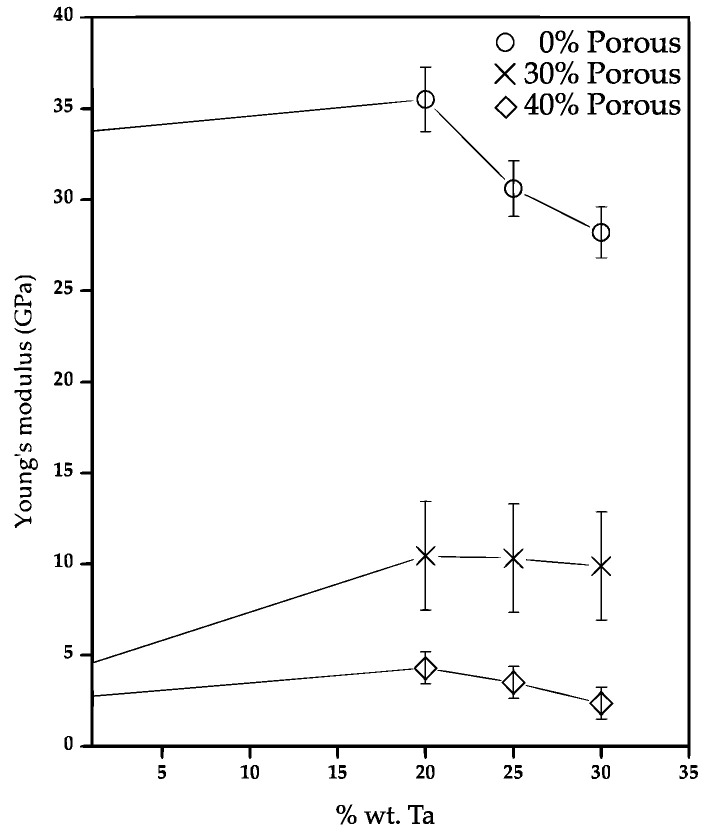
Young’s modulus as a function of the weight% of Ta for samples with and without additional porosity added.

The elastic modulus is estimated from the elastic behavior of all samples shown in Figure 11. It is found that the Young modulus is reduced with the Ta addition, although the value with the different quantities of Ta addition is around 30–35 GPa, see Figure 12. This value remains higher than the one required for bone applications (<18 GPa) [7]. This reduction is mainly due to two phenomena. The first one is due to the different Ti phases composing the TixTa alloys. As it was above discussed, the *β*-Ti became predominantly as the Ta addition increased up to 30 wt.%. The values of E*_α_* and E*_β_* 105 and 70 GPa were reported [58,59], thus, lower Young modulus is expected to obtain by the *β*-Ti stabilization. The second phenomenon is related to the residual porosity, which also increased as the Ta wt.% increased. The residual porosity in samples without pore formers was roughly estimated from SEM images as follow: 3%, 6%, 8% and 10% for samples with 0, 20, 25 and 30 wt.% of Ta, respectively. Therefore, the resulting reduction of the elastic modulus is a combination of both, in where the Ti30Ta sample shows the lower E because present larger porosity and *β*-Ti phase. Nonetheless, the reduction of the elastic modulus is not enough to be in the good range for bone applications.

On the contrary, the elastic modulus is highly reduced by the addition of pores, the sample without Ta shows a reduction of around 7 times from 34 to 4.5 GPa when 30 vol.% of salt particles are used. This reduction increases 11 times from 34 to 3 GPa for 40 vol.% of salt. The elastic modulus of composites with 30 vol.% of salt particles increased around twice to the one obtained for the porous Ti samples. However, similar values obtained for samples with different quantities of Ta. The addition of 40 vol.% of salt particles generates similar values of elastic modulus for composites and Ti samples. This suggests that the mechanical behavior driven by the porosity, with a minor effect of the Ta addition.

The elastic modulus of the *β*-Ti-Ta alloys manufacturing by casting reported in different works ranged from 65–100 GPa [23,46,54] being lower than the value reported for pure Ti (104 GPa). Nevertheless, these materials still represent high values of mechanical properties compared to the human bone ones who’s ranging from 0–18 GPa [7,58] depending on the type of bone, its function and porosity. Table 3 summarizes the Young Modulus reported for Ti-Ta alloys and bones. This demonstrates that stabilization of the *β*-Ti phase can reduce the elastic modulus but is not enough for the range needed in bone implants applications [59]. Therefore, the addition of pores is needed to obtain materials with elastic modulus values close to those required for use as a medical implant (0–18 GPa) [7]. For such effect, the Ti-xTa alloys with 30 and 40 vol.% of salt particles fabricated in this work shows good properties for both, compact and trabecular bones. The large pores add a great advantage for trabecular bones since the permeability plays a major role in allow passing the body fluids with the nutrients that promotes the bone growth through the metallic implant. For such case, the elastic modulus of 3 GPa is around 10 times larger than the one of such bones. Nonetheless, it is much lower to the one of fully dense alloys, which will improve the adaptability of the metallic implant.

## 4. Conclusions

Ti-xTa alloys with controlled porosity were successfully fabricated by solid state sintering. The diffusion kinetics of Ta into the Ti matrix was determined by dilatometry concluding the Ta diffuses in solid state during sintering which affects the transition phase from *α* to *β* by reducing the transition temperature. It was also concluded that the addition of large pores does not modify the diffusion kinetics, although the densification is increased by the pore deformation during sintering. The resulting microstructure shows *β*-Ti stabilization with the formation of *α′* martensite phase that is formed during cooling after sintering due to the cooling rate and the Ta diffusion into the Ti matrix. It is found that additional pores are needed for reducing the elastic modulus close to the values of the human bones, since the *β*-Ti stabilization is not enough. In addition, large pores as the one generated by the salt particles provide permeability values close to that of human bones, which will favor the body fluid to pass throughout the implant, thus improving the bone growth. It is concluded that the addition of 25 wt.% of Ta with 40 vol.% of salt particles generates the best materials for trabecular bone implants.

## Figures and Tables

**Table 1:** Green and sintered densities of samples with different quantities of Ta with and without additional porosity.

	Vol.% Pore Formers					
	0%		30%		40%	
%Ta	Green (g/cm^3^)	Sintered (g/cm^3^)	Green (g/cm^3^)	Sintered (g/cm^3^)	Green (g/cm^3^)	Sintered (g/cm^3^)
0	3.43	4.27	1.56	2.06	1.39	1.54
20	3.53	4.30	1.75	2.23	1.49	1.93
25	3.94	4.64	1.94	2.50	1.50	1.99
30	4.30	5.24	2.03	2.63	1.51	2.00

**Table 2:** Porosity and solid features of samples fabricated with different vol.% of salt particles.

Sample	Pore Volume Fraction (%)	Median Pore Size (µm)	Median Wall Size (µm)	Permeability Axial (10^−10^ m^2^)	Permeability Radial (10^−10^ m^2^)	Tortuosity
Ti/xTa30Salt	42.1	195	175	0.18	0.17	1.56
Ti/xTa40Salt	58.3	224	150	1.15	1.12	1.34

**Table 3:** Mechanical properties of Ti-Ta alloys and human bones reported in the literature.

Alloys		Young’s Modulus (GPa)
Cp-Ti [53,60]		103–105
Ti-20Ta [46,53,60]		83–84
Ti-30Ta [46,53,60]		69
Ti-50Ta [46,53,54]		77–93
**Bone** [7]		
Compact	Transverse	17.9 ± 3.9
	Longitudinal	10.1 ± 2.4
Trabecular	Vertebra	0.067 ± 0.045
	Tibia	0.445 ± 0.257
	Femur	0.441 ± 0.271

## Data Availability

The raw/processed data required to reproduce these findings cannot be shared at this time as the data also forms part of an ongoing study.
